# Mortality prediction with adjuvant tamoxifen in breast cancer: Machine learning-integrated explainable artificial intelligence and Bayesian model results

**DOI:** 10.5599/admet.3321

**Published:** 2026-04-29

**Authors:** Kannan Sridharan, Gowri Sivaramakrishnan

**Affiliations:** 1 Department of Pharmacology & Therapeutics, College of Medicine & Health Sciences, Arabian Gulf University, Manama, Kingdom of Bahrain; 2 Bahrain Defence Force Royal Medical Services, Riffa, Kingdom of Bahrain

**Keywords:** Selective estrogen receptor modulator, personalized medicine, extreme gradient boosting, hormonal therapy

## Abstract

**Background and purpose:**

Tamoxifen is a cornerstone of adjuvant endocrine therapy for breast cancer, yet significant inter-individual variability in treatment response and mortality exists. Identifying robust predictors of outcomes remains a critical need. This study integrated machine learning, explainable artificial intelligence (XAI) and Bayesian modelling to predict mortality and identify key prognostic factors in breast cancer patients receiving adjuvant tamoxifen.

**Experimental approach:**

We analysed data from 568 patients from the International Tamoxifen Pharmacogenomics Consortium database. The outcome was all-cause mortality, with predictors including age, race, menopausal status, tumour size, estrogen receptor status, radiation treatment, and CYP2D6 metabolizer status. Four algorithms, logistic regression, random forest, eXtreme Gradient Boosting (XGBoost) and support vector machine, were developed and validated. Model performance was assessed using accuracy and area under the receiver operating characteristic curve (AUC). SHapley Additive exPlanations (SHAP) analysis provided interpretability for the XGBoost model, and Bayesian logistic regression with weakly informative priors was employed for probabilistic inference.

**Key results:**

The overall mortality rate was 19.4 %. XGBoost demonstrated the highest discriminative ability (AUC 0.833; 95 % confidence interval: 0.725 to 0.941), while random forest exhibited superior sensitivity for identifying deceased patients (83.3 %). SHAP analysis revealed that white race, increased age, absence of radiation treatment, larger tumour size and the CYP2D6 poor metabolizer (PM/PM) genotype were associated with elevated mortality risk, whereas the extensive metabolizer (EM/EM) genotype was protective. Significant variability was observed in exploratory subgroup analyses, with the model achieving excellent discrimination in patients without radiation treatment (AUC 0.901) and those with the EM/PM genotype (AUC 0.956) but failing to identify any mortality events in the Caucasian subgroup. Bayesian logistic regression yielded comparable performance to frequentist methods (AUC 0.820), with tumour size emerging as a consistently strong predictor in partial dependence plots.

**Conclusion:**

Integrating machine learning with XAI and Bayesian approaches effectively identified key predictors of mortality in tamoxifen-treated breast cancer patients. However, marked heterogeneity in model performance across subgroups highlights the critical need for external validation and careful evaluation of algorithmic fairness before clinical implementation.

## Introduction

Breast cancer remains the most diagnosed malignancy among women worldwide, with a global age-standardized incidence rate of 46.3 *per* 100,000 population [1]. Endocrine therapy plays a pivotal role in the management of breast cancer, particularly in postmenopausal women, and tamoxifen, selective estrogen receptor modulator, is one of the most widely used agents in this context [2]. For decades, 5 years of adjuvant tamoxifen has been the standard of care for reducing both the incidence and recurrence of hormone-sensitive breast cancer [3]. Although tamoxifen primarily exerts its effects through estrogen receptor modulation, approximately 5 to 10 % of estrogen receptor-negative breast cancers have also demonstrated clinical response to the drug [4]. A recent systematic review and meta-analysis reported comparable mortality risks between tamoxifen and aromatase inhibitors, with a relative risk of 0.89 (95 % confidence interval (CI): 0.78 to 1.03) [5]. However, compared with tamoxifen, aromatase inhibitors have been associated with a higher incidence of arthralgia (relative risk (RR) = 1.55, 95 % CI: 1.20 to 1.99) and bone pain (RR = 1.31, 95 % CI: 1.05 to 1.62) [6].

Tamoxifen is a prodrug that requires metabolic conversion via the cytochrome P450 2D6 (CYP2D6) enzyme into its active metabolite, endoxifen, which is directly linked to both therapeutic efficacy and adverse effect profiles [7,8]. Despite its widespread use, the predictors of optimal response to tamoxifen therapy remain inadequately characterized. Previous studies have primarily focused on individual clinical or pharmacogenomic factors using conventional regression-based approaches, often yielding heterogenous and inconsistent findings regarding the role of CYP2D6 metabolizer status and demographic variables in tamoxifen outcomes [9]. Furthermore, these traditional methods are limited in their ability to capture complex, non-linear interactions among multiple predictors simultaneously. Consequently, a more comprehensive, integrative approach that can accommodate high-dimensional interactions and provide interpretable predictions of mortality risk in tamoxifen-treated patients is needed.

In recent years, machine learning algorithms (MLAs) have emerged as powerful tools in personalized medicine, enabling the identification of complex, non-linear relationships among predictor variables [10]. Nevertheless, the clinical applicability of MLAs is often constrained by their "black box" nature, wherein the rationale underlying model predictions remain opaque [11]. Explainable artificial intelligence (XAI) addresses this limitation by elucidating the contributing features and quantifying their influence on model outputs [12]. In the present study, we integrate MLA and XAI approaches to identify key predictors of tamoxifen response and mortality outcomes in patients with breast cancer.

## Experimental

### Study design and ethics

This study utilized a publicly available, open-access dataset from the International Tamoxifen Pharmacogenomics Consortium (ITPC), which aggregated data from 12 international sites on patients with breast cancer who received tamoxifen therapy [13]. As the analysis involved de-identified, anonymized data from a publicly accessible repository, ethics approval was not required.

### Study procedure

From the ITPC dataset, we included all patients with breast cancer who received tamoxifen. Patients were excluded if they had incomplete data for any of the following key variables: mortality outcome, age, race, menopausal status, maximum tumour dimension, estrogen receptor status, radiation treatment, or details on adjuvant therapy (specifically, whether they received a non-tamoxifen drug as the first adjuvant treatment or any additional adjuvant/chemotherapy). Cases with an unknown CYP2D6 genotype status were also excluded.

The primary outcome variable for this study was all-cause mortality. The following variables were selected as potential predictors based on clinical relevance: age; race (categorized as African American, Asian, Black, Caucasian, Korean, Hispanic and White); menopausal status (premenopausal, perimenopausal, postmenopausal); maximum tumour diameter; estrogen receptor status (present/absent); radiation treatment (received/ /not received); and CYP2D6 genotype, which was used to define metabolizer status.

CYP2D6 allele function and metabolizer phenotypes were defined according to standard criteria. Alleles were categorized as follows: poor metabolizer (*3, *4, *5, *6, *7, *8, *11, *12, *13, *14, *15, *16, *18, *19, *20, *38, *40, *42, *44, *56, 4XN), intermediate (9, *9XN, *10, *10XN, *17, *29, *37, *41, *41XN, 45, 46); extensive (1, *2, *2A, *33, *35, *39, 43) and ultrarapid (1XN, *2XN, *35XN, *39XN). Subsequently, patients were assigned a metabolizer status based on their diplotype: extensive (combinations of extensive (EM) and ultrarapid (UM) alleles: EM/EM, EM/UM, IM/UM, UM/UM), intermediate (combinations involving intermediate (IM) or poor (PM) alleles: EM/PM, IM/IM, IM/PM, PM/UM, EM/IM) and poor (PM/PM).

### Statistical methods section

#### Descriptive and comparative analyses

Baseline characteristics of the study cohort were summarized using descriptive statistics. Continuous variables (age and maximum tumour dimension) were reported as mean ± standard deviation, while categorical variables (race, menopausal status, estrogen receptor status, radiation treatment, CYP2D6 genotype, and metabolizer status) were presented as frequencies and percentages. For the outcome-stratified descriptive analysis, continuous variables were compared between patients who died and those who survived using the independent two-sample t-test (Welch's correction) for age and the Wilcoxon rank-sum test for tumour size, given the non-normal distribution of the latter. Categorical variables were compared using the chi-square test and Fisher's exact test with very small, expected counts.

#### Model development and validation framework

The dataset was randomly partitioned into training (80 %) and testing (20 %) sets using stratified sampling to preserve the proportion of the primary outcome in both subsets. All machine learning model development was performed exclusively on the training set. To ensure robust internal validation and prevent overfitting, hyperparameter tuning was conducted via 10-fold cross-validation on the training data. Final model performance was then evaluated on the held-out test set. To prevent data leakage, all preprocessing steps, including one-hot encoding of categorical variables and standardization of numerical variables, were fitted on the training data and then applied to the test set.

#### Machine learning algorithms

We developed and validated four MLAs: logistic regression with elastic net regularization, random forest, extreme gradient boosting (XGBoost), and support vector machine (SVM) with a radial kernel. Hyperparameter optimization for each algorithm was performed using a grid search approach with 10-fold cross-validation, selecting the parameters that maximized the area under the receiver operating characteristic curve (AUC).

#### Model performance assessment

Model performance was comprehensively evaluated on the test set using multiple metrics, including accuracy, sensitivity, specificity, precision, F1-score, Cohen's kappa and AUC. Corresponding 95 % CIs for these metrics were derived from bootstrap resampling with 1,000 iterations. Calibration was assessed using the Brier score (the mean squared difference between predicted probabilities and actual outcomes), log loss (cross-entropy loss), and the calibration slope, obtained by regressing the observed outcomes on the predicted probabilities (where a slope of 1 indicates perfect calibration).

### Feature importance and model interpretation

Feature importance was assessed using model-specific approaches: mean decrease in Gini impurity for random forest, gain for XGBoost, absolute coefficient values for logistic regression, and permutation importance for the SVM. Permutation importance was calculated by measuring the reduction in model accuracy and AUC after randomly shuffling the values of a single feature, holding all others constant. This process was repeated ten times per feature to ensure stability, and the average reduction in performance was reported.

To provide a unified and interpretable measure of feature effects, we applied SHAP (SHapley Additive exPlanations) analysis to the XGBoost model. SHAP values, grounded in cooperative game theory, quantify the marginal contribution of each feature to the prediction for an individual patient. The tree explainer algorithm, which leverages the tree structure for efficient computation, was used to generate SHAP values. Results were visualized using beeswarm plots to display the distribution of SHAP values for each feature, coloured by feature value, and summary bar plots showing the mean absolute SHAP value per feature with 95 % CIs derived from bootstrap resampling. To validate the consistency of feature importance, SHAP values were compared between concordant and discordant predictions using Wilcoxon rank-sum tests with Benjamini-Hochberg correction for multiple comparisons.

### Internal validation

Internal validity was further assessed using bootstrap resampling with 100 iterations. In each iteration, models were refit on a bootstrap sample of the training data and evaluated on the original test set. This process provided robust estimates of performance variability and helped quantify potential overfitting. All performance metrics from bootstrap validation were summarized as means with 95 % CIs based on the percentile method.

### Bayesian analysis

A Bayesian logistic regression model was implemented with weakly informative priors to provide probabilistic estimates of model parameters and predictions. The model was specified with normal priors cantered at zero with a scale of 2.5 for the regression coefficients and a normal prior with mean zero and scale of 5 for the intercept. The choice of weakly informative rather than strongly informative priors was deliberate and justified by the unique characteristics of the ITPC dataset. Unlike previous studies on tamoxifen response, which were conducted predominantly in homogeneous populations, the ITPC dataset is multinational and multiethnic, comprising Asian, Black, Caucasian, Hispanic, Korean, and White patients. Effect estimates from prior studies may not be directly transportable to this diverse population, and imposing strong informative priors could bias estimates away from true effects in under-represented groups. Weakly informative priors provide regularization without imposing strong substantive assumptions, allowing the data to inform estimates while maintaining the exploratory, hypothesis-generating nature of this study. Four Markov chain Monte Carlo chains were run with 2,000 iterations each, including a 1,000-iteration warmup period, yielding a total of 4,000 posterior draws for inference. Chain convergence was assessed using the potential scale reduction factor (*R*-hat, with values <1.1 indicating convergence) and by visual inspection of trace plots. Posterior predictive distributions were generated for the test set, and model performance metrics (AUC, accuracy, sensitivity, specificity and Brier score) were calculated with their 95 % credible intervals (CrI). Partial dependence plots were generated for all features by marginalizing over the joint distribution of other predictors, with uncertainty bands representing 95 % CrI derived from the posterior distribution.

### Exploratory subgroup analyses

To explore the generalizability and performance consistency of the best-performing model (XGBoost) across clinically relevant patient populations, we conducted subgroup analyses. Model performance metrics were assessed within strata defined by race, menopausal status, radiation treatment, CYP2D6 genotype, and meta-bolizer status, using the held-out test set to ensure independence from model training. These exploratory analyses are intended to generate hypotheses regarding potential heterogeneity in model performance and should not be interpreted as definitive comparisons. Performance of other machine learning algorithms across subgroups was not examined in this exploratory study but represents an important direction for future research.

### Statistical significance and software

All statistical tests were two-tailed, and a *p*-value of ≤0.05 was considered statistically significant. All analyses were performed using *R* (version 4.5.2) [14].

## Results

### Study participants

A total of 4,973 patients from the ITPC database were initially considered for this study. After applying the predefined inclusion and exclusion criteria, 568 patients were included in the final analysis. The detailed reasons for exclusion are provided in Supplementary material Table S1. The baseline demographic and clinical characteristics of the study cohort are summarized in Table 1.

**Table 1.** Baseline characteristics of included patients (*n* = 568)

Characteristic		Values
Height, cm		161.3 ± 6.3
Weight, kg		70.2 ± 14.2
Age at diagnosis, year		61.3 ± 11.5
Female gender		567 (99.8 %)
Race	African American	3 (0.5 %)
	Asian	96 (16.9 %)
	Black	15 (2.6 %)
	Caucasian	183 (32.2 %)
	Korean	25 (4.4 %)
	Latina/Hispanic	2 (0.4 %)
	White	244 (43 %)
Laterality of tumour (*n* = 415)	Bilateral	1 (0.2 %)
	Unilateral	414 (99.8 %)
Menopause tatus	Perimenopausal	8 (1.4 %)
	Postmenopausal	455 (80.1 %)
	Premenopausal	105 (18.5 %)
Nottingham grade (*n* = 407)	1	115 (28.3 %)
	2	213 (52.3 %)
	3	79 (19.4 %)
Estrogen receptor positive status		553 (97.4 %)
Received radiation treatment		269 (47.4 %)
CYP2D6 genotype	EM/EM	253 (44.5 %)
	EM/IM	97 (17.1 %)
	EM/PM	138 (24.3 %)
	EM/UM	3 (0.5 %)
	IM/IM	31 (5.5 %)
	IM/PM	23 (4.0 %)
	PM/PM	23 (4.0 %)
CYP2D6 metabolizer status	Extensive	256 (45.1 %)
	Intermediate	289 (50.9 %)
	Poor	23 (4.0 %)

Numerical variables are represented as mean ± SD, while categorical variables as contribution, %.

The overall mortality rate in the cohort was 19.4 % (*n* = 110). Table 2 presents a comparison of characteristics stratified by outcome status (alive *vs.* dead), along with statistical significance tests for each variable. Patients who died were significantly older (70.9 ± 9.0 *vs.* 59.0 ± 10.9 years; *p* <0.001), had larger tumours (24.1 ± 13.6 *vs.* 18.7 ± 10.8 mm; *p* <0.001), were less likely to have received radiation therapy (15.5 *vs.* 55.0 %; *p* <0.001), and were more predominantly White (88.2 *vs.* 32.1 %; *p* <0.001) compared to those who survived. No significant differences were observed between the two groups with respect to estrogen receptor status (*p* = 0.324), CYP2D6 genotype distribution (*p* = 0.196), or metabolizer status categories (*p* = 0.346).

**Table 2.** Comparison of Characteristics Between Those Alive and Dead.

Characteristic		Alive (*n* = 458)	Dead (*n* = 110)	*p*-value
Age at diagnosis, year		59 ± 10.9	70.9 ± 9	<0.001*
Tumour size, mm		18.7 ± 10.8	24.1 ± 13.6	<0.001*
Race	African American	3 (0.7 %)	0 (0 %)	<0.001*
	Asian	96 (20.9 %)	0 (0 %)	
	Black	10 (2.2 %)	5 (4.5 %)	
	Caucasian	176 (38.4 %)	7 (6.4 %)	
	Korean	24 (5.2 %)	1 (0.9 %)	
	Latina/Hispanic	2 (0.4 %)	0 (0 %)	
	White	147 (32.1 %)	97 (88.2 %)	
Menopausal status	Perimenopausal	8 (1.7 %)	0 (0 %)	<0.001*
	Postmenopausal	346 (75.5 %)	109 (99.1 %)	
	Premenopausal	104 (22.7 %)	1 (0.9 %)	
Estrogen receptor status	Negative	14 (3.1 %)	1 (0.9 %)	0.324
	Positive	444 (96.9 %)	109 (99.1 %)	
Radiation therapy	No	206 (45 %)	93 (84.5 %)	<0.001*
	Yes	252 (55 %)	17 (15.5 %)	
CYP2D6 genotype	Extensive	202 (44.1 %)	54 (49.1 %)	0.1959
	Intermediate	240 (52.4 %)	49 (44.5 %)	
	Poor	16 (3.5 %)	7 (6.4 %)	
Metabolizer status	EM/EM	200 (43.7 %)	53 (48.2 %)	0.3463
	EM/IM	84 (18.3 %)	13 (11.8 %)	
	EM/PM	110 (24 %)	28 (25.5 %)	
	EM/UM	2 (0.4 %)	1 (0.9 %)	
	IM/IM	28 (6.1 %)	3 (2.7 %)	
	IM/PM	18 (3.9 %)	5 (4.5 %)	
	PM/PM	16 (3.5 %)	7 (6.4 %)	

*Statistically significant. Numerical variables are represented as mean ± SD, while categorical variables as contribution, %. Numerical tests were compared using independent t test or Wilcoxon-signed rank sum test while categorical variables using either *χ*^2^ test or Fisher’s exact probability test.

### Machine learning analysis

#### Cohort characteristics

The training (*n* = 455) and testing (*n* = 113) cohorts were well-balanced, with no significant differences in the distribution of mortality outcomes [90 (19.8 %) *vs.* 20 (17.7 %), respectively; *p* = 0.71]. Except for estrogen receptor status, all other baseline characteristics were similarly distributed between the two cohorts (Table 3).

**Table 3.** Comparison of characteristics between training and testing cohorts.

Variable	Category	Training cohort (*n* = 455)	Testing cohort (*n* = 113)	*p* value
Age, year		61.3 ± 11.8	61.2 ± 10.6	0.92
Tumor size, mm		19.7 ± 11.8	19.7 ± 10.5	0.98
Race	African American	3 (0.7 %)	0	0.62
	Asian	78 (17.1 %)	18 (15.9 %)	
	Black	12 (2.6 %)	3 (2.7 %)	
	Caucasian	150 (33 %)	33 (29.2 %)	
	Korean	16 (3.5 %)	9 (8 %)	
	Latina/Hispanic	2 (0.4 %)	0	
	White	194 (42.6 %)	50 (44.2 %)	
Menopause	Perimenopausal	6 (1.3 %)	2 (1.8 %)	0.60
	Premenopausal	87 (19.1 %)	93 (82.3 %)	
	Postmenopausal	362 (79.6 %)	18 (15.9 %)	
Positive estrogen receptor status		440 (96.7 %)	113 (100 %)	0.05*
Radiation treatment present		216 (47.5 %)	53 (46.9 %)	1.00
CYP2D6 genotype	EM/EM	205 (45.1 %)	48 (42.5 %)	0.13
	EM/IM	70 (15.4 %)	27 (23.9 %)	
	EM/PM	117 (25.7 %)	21 (18.6 %)	
	EM/UM	1 (0.2 %)	2 (1.8 %)	
	IM/IM	24 (5.3 %)	7 (6.2 %)	
	IM/PM	19 (4.2 %)	4 (3.5 %)	
	PM/PM	19 (4.2 %)	4 (3.5 %)	
Metabolizer status	Extensive	206 (45.3 %)	50 (44.2 %)	0.97
	Intermediate	230 (50.5 %)	59 (52.2 %)	
	Poor	19 (4.2 %)	4 (3.5 %)	

*Statistically significant. Numerical variables are represented as mean ± SD, while categorical variables as contribution, %. Numerical tests were compared using independent t test while categorical variables using either *χ*^2^ test or Fisher’s exact probability test.

#### Model performance

**Figure 1.**
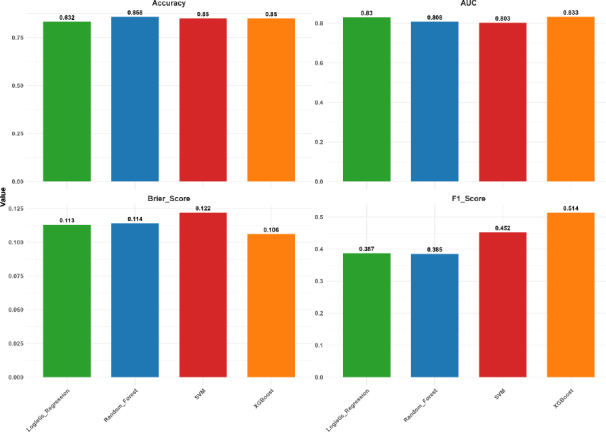
Performance metrics of machine learning algorithms. Vertical bars depict the values of respective performance metrics for individual machine learning algorithms

**Figure 2.**
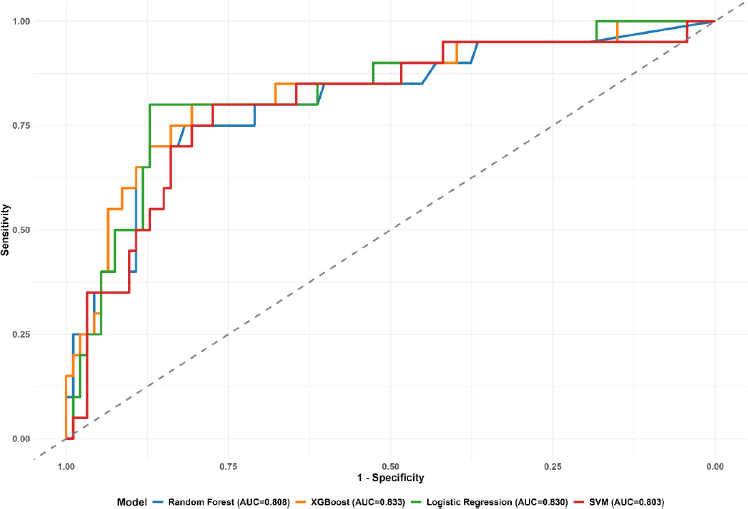
ROC plots of MLAs

All four machine learning algorithms demonstrated comparable overall performance (Figure 1). Random forest achieved the highest accuracy (0.858; 95 % CI: 0.78, 0.917), followed closely by XGBoost (0.85; 95 % CI: 0.77, 0.91) and SVM (0.85; 95 % CI: 0.77, 0.91). However, XGBoost yielded the highest AUC (0.833; 95 % CI: 0.725, 0.941), followed by logistic regression (0.83; 95 % CI: 0.725, 0.935) (Figure 2).

Examination of the confusion matrices (Table S2) revealed distinct prediction patterns. XGBoost correctly identified 88.8 % of patients who were alive but correctly predicted only 60 % of those who died. In contrast, random forest correctly identified 86 % of alive patients and demonstrated superior sensitivity, correctly predicting 83.3 % of deceased patients.

**Figure 3.**
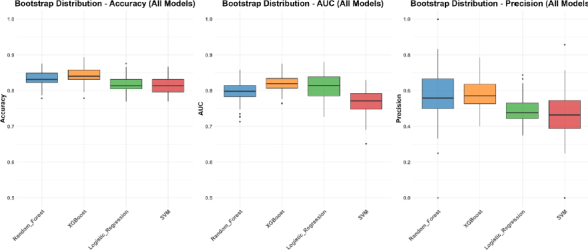
Bootstrap validation of key performance metrics of MLAs. Vertical bars depict the values of respective performance metrics for individual machine learning algorithms

Bootstrap validation confirmed the stability of these findings. XGBoost consistently provided strong performance metrics with relatively low variability across bootstrap samples (Figure 3 and Table S3).

### Feature Importance

Feature importance was successfully extracted from all models except SVM. Across the random forest, XGBoost, and logistic regression models, White race and age consistently emerged as the two most important predictors of mortality (Supplementary material Figure S1).

### Shapley additive explanations analysis

The SHapley Additive exPlanations (SHAP) analysis, applied to the XGBoost model to provide interpretable insights, revealed the direction and magnitude of feature contributions to mortality risk (Supplementary material Figure S2). Higher SHAP values indicate a greater contribution toward predicting mortality. White race, increased age, absence of radiation treatment, larger tumour size, and the poor metabolizer genotype (PM/PM) for CYP2D6 were associated with positive SHAP values, indicating their contribution to an increased predicted risk of mortality. Conversely, the extensive metabolizer genotype (EM/EM) was associated with negative SHAP values, indicating a protective effect (*i.e.* contribution toward predicting survival).

A comparative analysis of SHAP values between concordant (correctly predicted) and discordant (incorrectly predicted) cases revealed significant differences in how certain features influenced model predictions (Supplementary material Table S4). Age demonstrated a notable disparity, with a negative mean SHAP value in the concordant group (contributing to correct survival predictions) but a positive mean SHAP value in the discordant group (contributing to incorrect mortality predictions), a difference that was statistically significant. Similarly, racial categories, specifically Asian, Black, and White race, showed significant differences in their SHAP values between concordant and discordant groups. For instance, White race had a strongly negative mean SHAP in the concordant group but a positive mean SHAP in the discordant group, highlighting a potential source of predictive inconsistency. In contrast, features such as tumour size, radiation treatment, and most CYP2D6 genotype categories did not exhibit significant differences, implying that their contributions to model predictions are more stable, regardless of prediction accuracy. However, the PM/PM genotype did show a statistically significant difference, though its mean SHAP values were very small in both groups, suggesting a subtle but detectable shift in its impact.

An examination of the five patients with the highest predicted risk of mortality (*i.e.* the "worst" predictions) revealed that older age, White race, absence of radiation treatment, and larger tumour size were consistently associated with positive SHAP values, driving the high-risk predictions (Supplementary material Figure S3). Conversely, in the five patients with the lowest predicted risk (the "best" predictions), strongly negative SHAP values were observed for these same variables, contributing to the prediction of survival (Supplementary material Figure S4).

### Bayesian logistic regression analysis

The Bayesian logistic regression model demonstrated performance metrics comparable to its frequentist counterpart (accuracy: 0.805; 95 % CrI: 0.720 to 0.874; AUC: 0.82; 95 % CrI: 0.720 to 0.920; Supplementary material Figure S5). Other key performance metrics of the Bayesian analysis are summarized in Table 4.

**Table 4.** Key performance metrics of Bayesian analysis

Performance metric	Value
Sensitivity	0.444
Specificity	0.874
Precision	0.4
F1 score	0.421
Balanced accuracy	0.659
Brier score	0.128
Log loss	0.438
Calibration slope	0.472
R-hat range	1-1.006
Parameters with R-hat >1.1	None
Convergence status	Successful
LOO information criterion	
ELPD	-156.12
p_Loo	18.95
LOOIC	312.25

ELPD = Expected log predictive density, p_Loo: Effective number of parameters and LOOIC = Leave-one-out information criterion.

All R-hat values were ≤1.01, indicating successful chain convergence, and effective sample sizes (ESS) were >1,000 for all parameters, suggesting adequate posterior sampling. The mean width of the prediction uncertainty interval for mortality in the test cohort was 0.23, reflecting the model's precision. Examination of Bayesian partial dependence plots revealed that larger tumour size was associated with a clear, steady increase in the probability of mortality (Supplementary material Figure S6). Among demographic factors, age demonstrated a slight positive association with mortality risk.

### Exploratory sub-group analysis

The performance of the XGBoost model varied substantially across clinically relevant patient subgroups (Table 5). Particularly, the model demonstrated exceptional performance for the Caucasian subgroup in terms of specificity (1.00) and accuracy (0.92). The model also showed a strong discriminative ability for patients who did not receive radiation treatment, with an AUC of 0.901 and a well-calibrated slope of 1.492, suggesting reliable probability estimates. Among CYP2D6 genotype subgroups, the model achieved its highest discriminative performance (AUC = 0.956) in patients with the EM/PM genotype, though sensitivity remained modest at 0.50, highlighting a trade-off between identifying true positives and overall classification accuracy.

**Table 5.** Comparison of performance metrics of XGBoost model results across sub-groups

Subgroup	Category	Accuracy	AUC (95 % CI)	Sensitivity	Specificity	F1 Score	Brier Score	Calibration Slope
Race	White	0.74	0.801 (0.670-0.933)	0.562	0.824	0.581	0.167	1.174
	Caucasian	0.909	0.522 (0.113-0.932)	0	1	NE	0.088	0.145
Postmenopausal status		0.817	0.797 (0.671-0.922)	0.45	0.918	0.514	0.128	0.715
Radiation	Yes	0.887	0.692 (0.458-0.925)	0	0.979	NE	0.093	0.332
Radiation	No	0.817	0.901 (0.825-0.976)	0.6	0.889	0.621	0.117	1.492
CYP2D6 genotype	EM/EM	0.854	0.797 (0.606-0.988)	0.5	0.947	0.588	0.12	0.708
	EM/PM	0.857	0.956 (0.872-1.000)	0.5	0.941	0.571	0.074	1.684
	IM/IM	0.714	0.800 (0.340-1.000)	0	1	NE	0.191	0.953
	EM/IM	0.852	0.833 (0.632-1.000)	0.333	0.917	0.333	0.1	0.723
Metabolizer status	Extensive	0.86	0.802 (0.613-0.992)	0.5	0.95	0.588	0.116	0.728
	Intermediate	0.847	0.884 (0.769-0.999)	0.4	0.939	0.471	0.096	0.884

NE-Not estimable

Beyond the quantitative performance metrics reported in Table 5, several patterns emerged that indicate fundamental issues in the model's classification capability. Particularly, the model achieved a sensitivity of 0 in Caucasian, patients who received radiation treatment, and those with IM/IM genotype subgroups, indicating the model’s inability to identify any deceased individuals within these groups despite reasonable overall accuracy along with non-estimable F1 scores. These patterns suggest that the model has learned to predict survival almost exclusively, effectively defaulting to the majority class in certain strata. Such behaviour cannot be captured by accuracy or AUC alone, highlighting the necessity of disaggregated performance evaluation across clinically meaningful subgroups.

## Discussion

### Statement of key findings

The present study leveraged machine learning, XAI, and Bayesian modelling to identify predictors of mortality in breast cancer patients receiving adjuvant tamoxifen. Among the four algorithms evaluated, XGBoost demonstrated the highest discriminative ability. SHAP analysis revealed that White race, increased age, absence of radiation therapy, larger tumour size, and the CYP2D6 poor metabolizer (PM/PM) genotype were associated with an elevated risk of mortality, whereas the extensive metabolizer (EM/EM) genotype conferred a protective effect. Notably, the influence of demographic factors such as age and race on model predictions differed significantly between correctly and incorrectly classified cases, suggesting potential sources of bias or complexity in how these variables interact with outcomes. Bayesian logistic regression provided probabilistic estimates with performance comparable to frequentist methods, and partial dependence plots confirmed tumour size as a consistently strong predictor. Exploratory subgroup analyses revealed marked performance heterogeneity, with the model demonstrating excellent discrimination in patients who did not receive radiation and those with the EM/PM genotype but failing to identify any mortality events in the Caucasian subgroup. These findings underscore the potential of integrating ML and XAI to uncover complex predictor-outcome relationships in tamoxifen-treated patients, while also highlighting critical challenges related to model generalizability and fairness across diverse clinical and demographic subgroups.

### Comparison with existing literature

Our findings demonstrate that ensemble tree-based methods, particularly XGBoost and random forest, achieved robust predictive performance, with XGBoost yielding the highest discriminative ability and random forest demonstrating superior sensitivity for identifying deceased patients. The application of SHAP analysis provided granular interpretability, revealing that White race, increased age, absence of radiation treatment, larger tumour size, and the CYP2D6 poor metabolizer genotype were associated with elevated mortality risk, while the extensive metabolizer genotype conferred a protective effect. These findings align with established clinical knowledge while offering novel insights into the complex interplay of pharmacogenomic and demographic factors in tamoxifen response. Further, the direction of associations observed in the unadjusted stratified analysis aligns with the SHAP-derived feature effects: older age, larger tumour size, absence of radiation, White race, and poor metabolizer status were all associated with higher mortality in both the descriptive comparison and the XGBoost model's predictions, confirming consistency between the raw data patterns and the machine learning interpretations.

The emergence of tumour size as a consistently strong predictor across both machine learning and Bayesian models is clinically expected yet methodologically reassuring. Larger tumour diameter has long been recognized as a poor prognostic factor in breast cancer, correlating with higher pathological stage, increased likelihood of nodal involvement, and greater metastatic potential [15]. The monotonic positive relationship between tumour size and mortality probability observed in our Bayesian partial dependence plots provides face validity for our modelling approach and confirms that the algorithms successfully captured this well-established clinical relationship. Similarly, the association between absence of radiation treatment and increased mortality risk likely reflects underlying disease characteristics is like the finding from a previous study where the incidence of local relapse with tamoxifen with irradiation was reported to be 0.6 % compared to 7.7 % with only tamoxifen [16]. However, radiation is typically omitted in patients with favourable prognosis or early-stage disease following breast-conserving surgery and so confounding by indication cannot be excluded.

The role of demographic factors, particularly age and race, in our predictive models warrants careful consideration. Increased age emerged as an important predictor of mortality, which may reflect competing risks, age-related differences in tumour biology, or variations in treatment tolerance and completion [17]. Another study that assessed breast density with tamoxifen revealed that in women aged 45 years or younger at entry, the net reduction with tamoxifen was 13.4 % (95 % CI = 8.6 to 18.1), whereas in women older than 55 years, it was 1.1 % (95 % CI = -3 to 5.1) [18]. Another factor that was not captured in the ITPC study was compliance. This is important as a study amongst 961 women on tamoxifen revealed that 49 % discontinued before the completion of 5 years and rate of discontinuation was more likely to be aged 75 to less than 80 years (hazard ratio (HR) = 1.41; 95 % CI 1.06 to 1.87), and amongst those aged ≥ 80 years (HR = 2.02; 95 % CI 1.53 to 2.66) compared to <70 years [19].

The findings on the impact of race, particularly the White race as a mortality predictor is more complex and potentially concerning. While this finding may reflect genuine biological or socioeconomic differences, it may also indicate ascertainment bias, given that the ITPC dataset predominantly comprises patients of European descent. The SHAP analysis revealed that White race contributed positively to mortality predictions, a finding that must be interpreted cautiously to avoid reinforcing potentially spurious associations. Notably, the comparative analysis of SHAP values between concordant and discordant predictions revealed that both age and race exhibited significantly different influences depending on whether the model's prediction was correct or incorrect. This observation suggests that the model's reliance on these demographic variables may be unstable or that important effect modifiers are missing from the analysis, leading to inconsistent performance. Studies have shown that Black population were more often reported with treatment discontinuation and toxicity risk with endocrine therapies for breast cancer compared to White population [20]. Additionally, race was not observed to influence breast density when followed up for a period of approximately 3 years on endocrine therapy [21].

The importance of CYP2D6 metabolizer status across multiple modelling approaches reinforces the biological plausibility of our findings. Tamoxifen is a prodrug that requires enzymatic conversion via CYP2D6 to its active metabolite, endoxifen, which is primarily responsible for its therapeutic effects [7,8]. Our observation that the poor metabolizer phenotype (PM/PM) was associated with increased mortality risk corroborates prior evidence linking reduced CYP2D6 activity to inferior clinical outcomes [22]. Conversely, the protective effect associated with the extensive metabolizer phenotype (EM/EM) supports the hypothesis that adequate metabolic capacity is essential for optimal tamoxifen efficacy as evidenced by reduction in progression-free and disease-free survival [23]. These findings have potential clinical implications, suggesting that genotyping for CYP2D6 prior to initiating tamoxifen therapy could identify patients at heightened risk of treatment failure who might benefit from alternative endocrine strategies or closer monitoring.

The substantial heterogeneity observed in subgroup model performance is both illuminating and cautionary. The XGBoost model achieved excellent discriminative ability in patients who did not receive radiation treatment and those with the EM/PM CYP2D6 genotype, suggesting that in well-defined clinical or genetic subgroups, machine learning approaches can provide reliable risk stratification. However, the model's complete inability to identify any mortality events in the Caucasian subgroup, despite high overall accuracy and specificity, exemplifies the limitations of aggregate performance metrics. This dissociation between accuracy and sensitivity highlights the risk of deploying models that appear performant on the surface but fail catastrophically in critical subsets of patients. Such findings underscore the necessity of rigorous subgroup evaluation to ensure that predictive tools do not inadvertently exacerbate healthcare disparities.

The finding that the model achieved zero sensitivity in certain subgroups despite reasonable overall accuracy underscores a critical limitation of relying on aggregate performance metrics. As observed in the Caucasian subgroup, an accuracy of 0.909 and specificity of 1 initially appear impressive; however, these values simply reflect the model's success in predicting the majority class (survival) while completely failing to identify any mortality events. This pattern, sometimes termed "accuracy paradox" or "majority class bias", is particularly problematic in clinical contexts where the cost of missing a true positive (a patient who dies) is substantially higher than the cost of a false positive. The presence of non-estimable F1 scores further confirms that the model is not merely underperforming but is fundamentally incapable of discriminating mortality risk in certain patient strata. These observations reinforce the necessity of reporting disaggregated performance metrics and, more importantly, of scrutinizing model behaviour in each subgroup before any consideration of clinical deployment. It is important to note that these subgroup analyses were exploratory in nature and were performed only for the XGBoost model. Other algorithms evaluated in this study, logistic regression, random forest, and SVM, may exhibit different patterns of subgroup performance. A comprehensive comparison of subgroup-specific metrics across all algorithms was beyond the scope of this exploratory study but should be prioritized in future externally validated cohorts.

The observed class imbalance warrants explicit consideration when interpreting model performance metrics. Machine learning algorithms trained on imbalanced data tend to favour the majority class (alive), achieving high accuracy and specificity at the expense of sensitivity. This phenomenon likely explains several findings in our study, including the model's high specificity but poor or zero sensitivity in subgroups such as Caucasian patients and those who received radiation therapy. The stratified descriptive analysis confirms that the unadjusted associations between predictors and mortality are largely consistent with the direction of effects identified by SHAP analysis and machine learning feature importance, providing reassurance that the model has not learned spurious correlations. However, readers should interpret sensitivity and F1 scores with caution given the class imbalance, as these metrics are more sensitive to minority class performance than accuracy or AUC. Future work should consider techniques such as class weighting, synthetic oversampling, or cost-sensitive learning to mitigate the effects of imbalance.

Our Bayesian logistic regression analysis complements the machine learning findings by providing probabilistic estimates with associated uncertainty. The comparable performance between Bayesian and frequentist approaches suggests that the additional complexity of Bayesian modelling may not always translate to improved point predictions, but the value lies in the quantification of prediction uncertainty [24]. The mean prediction uncertainty width of 0.23 for mortality probabilities provides clinicians with a sense of confidence around individual risk estimates, potentially supporting more nuanced treatment decisions. Furthermore, Bayesian partial dependence plots confirmed the monotonic relationship between tumor size and mortality while revealing a more modest association for age, providing probabilistic confirmation of the relationships identified through SHAP analysis.

The integration of XAI represents a significant advancement over traditional "black box" machine learning approaches in medical research. By quantifying the marginal contribution of each feature to individual predictions, SHAP analysis bridges the gap between predictive performance and clinical interpretability. This transparency is essential for building clinician trust and facilitating the translation of computational models into clinical practice. In our study, SHAP values not only identified important predictors but also revealed the direction of their effects and enabled comparison between correctly and incorrectly classified cases, offering insights into potential model limitations that would otherwise remain hidden. This methodological approach aligns with the growing recognition that interpretability is not merely a desirable adjunct to predictive modelling but a fundamental requirement for responsible AI in healthcare [25]. However, given the modest predictive performance and limited specificity demonstrated in this study, any discussion of clinical implementation must be preceded by substantial improvements in model accuracy [26]. In a real-world clinical setting, an XAI-enhanced decision support tool could be integrated into existing electronic health record systems or breast cancer clinic workflows [27].

### Strengths, limitations and way forward

This study possesses several notable strengths. It leverages a multi-institutional, international dataset, which enhances the generalizability of the findings compared to single-centre analyses. Furthermore, the integration of traditional MLAs with XAI (SHAP) and Bayesian modelling represents a robust methodological approach, allowing us not only to predict mortality but also to interpret the complex, non-linear contributions of individual predictors and quantify uncertainty in our estimates. The identification of key predictors, such as CYP2D6 metabolizer status and the interaction of demographic factors, provides clinically plausible insights that align with the existing pharmacogenomic literature.

However, the findings must be interpreted within the context of several limitations. First, the substantial number of patients excluded due to missing data, while necessary for methodological rigor, may introduce selection bias and limit the sample size for certain subgroup analyses, particularly among rare racial categories or CYP2D6 genotypes. Second, the dataset lacked information on other potential confounders, such as compliance with the tamoxifen regimen, concurrent medications that may inhibit CYP2D6 activity, hormone receptor positivity levels, or specific causes of death, all of which could influence the observed associations. Third, while internal validation was rigorous, the lack of external validation in an independent, contemporary cohort limits the immediate clinical applicability of our models. Fourth, the fundamental classification failures observed in several subgroups, including zero sensitivity in the Caucasian and radiation-treated groups, indicate that the model has learned a decision boundary that disproportionately favours predicting survival, a pattern that requires further investigation and correction before any clinical application could be considered. Furthermore, subgroup performance was evaluated only for the XGBoost model; other machine learning algorithms may demonstrate different patterns of classification success or failure across patient strata. The exploratory nature of these subgroup analyses means they should be interpreted as hypothesis-generating rather than confirmatory. Additionally, the moderate class imbalance in our cohort may have biased the machine learning models toward predicting the majority class, potentially contributing to the poor sensitivity observed in several subgroups; future studies should employ imbalance mitigation techniques to address this limitation.

For clinicians, these findings reinforce the importance of considering CYP2D6 genotype and tumor characteristics in the context of overall patient demographics when assessing risk. However, the variability in model performance across subgroups, particularly the poor sensitivity in Caucasian patients, serves as a critical cautionary tale against the blind application of "one-size-fits-all" predictive models. For researchers, the significant disparity in feature importance between concordant and discordant predictions highlights the need for deeper investigation into model bias and fairness. The way forward should prioritize external validation in large, prospective, and diverse cohorts. Future studies should also aim to integrate a wider array of variables, including detailed treatment adherence data, lifestyle factors, and genomic markers, into these models. Ultimately, the goal is to refine these tools to ensure they are not only accurate but also equitable and generalizable across the full spectrum of patients with breast cancer.

## Conclusions

In conclusion, this study dsemonstrates that MLAs, particularly XGBoost and random forest, can effectively predict mortality in breast cancer patients receiving adjuvant tamoxifen, with model interpretability enhanced through SHAP analysis and Bayesian approaches. Our findings confirm the prognostic importance of established clinical factors such as age, tumour size, and radiation treatment, while also highlighting the significant role of CYP2D6 metabolizer status and revealing complex influences of demographic variables like race that warrant further investigation. The observed heterogeneity in model performance across patient subgroups, identified through exploratory analyses, underscores both the promise and the peril of applying artificial intelligence in personalized oncology: while these tools can uncover nuanced predictor-outcome relationships, their clinical deployment requires rigorous validation to ensure accuracy and fairness across diverse populations. Future research must focus on external validation in prospective, multi-ethnic cohorts and the integration of additional clinically relevant variables to refine these predictive models, with the goal of equipping clinicians with reliable, interpretable tools to guide treatment decisions and improve outcomes for all patients undergoing tamoxifen therapy.

## Supplementary material

Additional Tables and Figures are available at https://pub.iapchem.org/ojs/index.php/admet/article/view/3321.


