# Supplementary material for: Mortality prediction with adjuvant tamoxifen in breast cancer: Machine learning-integrated explainable artificial intelligence and Bayesian model results
Source: ADMET DMPK. 2026 Apr 29;14:3321. doi: 10.5599/admet.3321 (PMC13147516; doi:10.5599/admet.3321)
Supplement: Supplementary file 1 [file ADMET-14-3321-S1.pdf]

ADMET & DMPK **14** (2026) S3321

Open Access : ISSN : 1848-7718
http://www.pub.iapchem.org/ojs/index.php/admet

Supplementary material to

Mortality prediction with adjuvant tamoxifen in breast cancer: Machine learning-integrated explainable artificial intelligence and Bayesian model results

Kannan Sridharan[1,*] and Gowri Sivaramakrishnan[2]

[1]*Department of Pharmacology & Therapeutics, College of Medicine & Health Sciences, Arabian Gulf University, Manama, Kingdom of Bahrain*
[2]*Bahrain Defence Force Royal Medical Services, Riffa, Kingdom of Bahrain*

ADMET & DMPK **14** (2026) 3321; https://doi.org/10.5599/admet.3321

Table S1. Reasons for exclusion

Reasons	Number of patients
Total number of patients in the ITPC dataset	4973
No data regarding death or alive status	6
No data on race	2583
No data on age	85
No data on menopause status	829
No data on maximum dimension of tumor	137
No data on estrogen receptor status	38
No data on radiation treatment	186
Non-tamoxifen adjuvant first	58
Additional adjuvant therapy	220
Adjuvant chemotherapy	276
No data on CYP2D6 status	6
Total included	568

Table S2. Confusion matrix of prediction of outcomes by MLAs

Predicted	Alive	Dead	Total	Predicted rate of alive, %	Predicted rate of dead, %	Model
Alive	92	15	107	86.00	14.00	Random forest
Dead	1	5	6	16.70	83.30	
Alive	87	11	98	88.80	11.20	XGBoost
Dead	6	9	15	40.00	60.00	
Alive	88	14	102	86.30	13.70	Logistic regression
Dead	5	6	11	45.50	54.50	
Alive	89	13	102	87.30	12.70	SVM
Dead	4	7	11	36.40	63.60	

Table S3. Comparison of accuracies and AUCs of amongst test cohorts and bootstrap validation

Model	Test Accuracy	Test Accuracy CI	Bootstrap Accuracy	Bootstrap Accuracy CI	Test AUC	Test AUC CI	Bootstrap AUC	Bootstrap AUC CI
Random forest	0.858	0.780 to 0.917	0.834	0.796 to 0.867	0.808	0.693 to 0.923	0.797	0.731 to 0.840
XGBoost	0.85	0.770 to 0.910	0.84	0.796 to 0.885	0.833	0.725 to 0.941	0.822	0.781 to 0.866
Logistic regression	0.832	0.750 to 0.896	0.818	0.779 to 0.858	0.83	0.725 to 0.935	0.81	0.738 to 0.870
SVM	0.85	0.770 to 0.910	0.817	0.779 to 0.854	0.803	0.689 to 0.917	0.766	0.693 to 0.814

Table S4. Comparison of SHAP values between concordant and discordant groups

Feature	Mean SHAP in concordant group	Mean SHAP in discordant group	Mean absolute SHAP in concordant group	Mean absolute SHAP in discordant group	Adjusted *p*-values
Age	-0.44	0.19	0.76	0.59	0.01*
Tumor size	-0.12	-0.06	0.35	0.36	0.80
Asian race	-0.17	0.05	0.24	0.05	0.01*
Black race	-0.06	0.02	0.12	0.11	0.01*
White race	-0.37	0.44	1.06	1.08	0.01*
Postmenopausal status	-0.04	0.00	0.05	0.00	0.07
No radiation treatment	-0.21	-0.08	0.47	0.35	0.89
CYP2D6 EM/EM	0.00	0.01	0.03	0.04	0.25
CYP2D6 Genotype EM/IM	0.04	0.02	0.08	0.05	0.48
CYP2D6 Genotype EM/PM	0.00	-0.01	0.02	0.02	0.52
CYP2D6 Genotype PM/PM	-0.01	0.00	0.03	0.04	0.01*

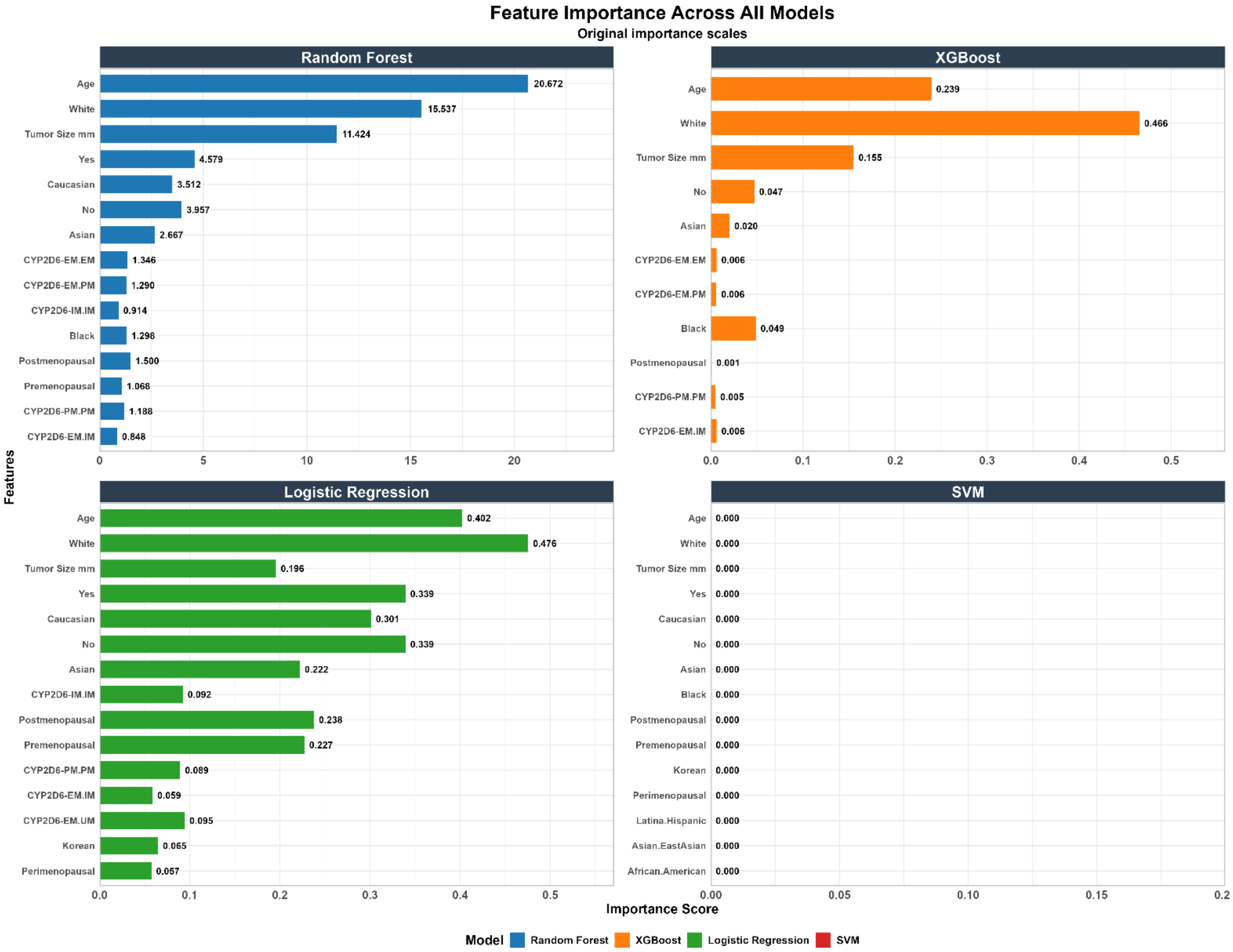

Figure S1. Feature importance across MLA models

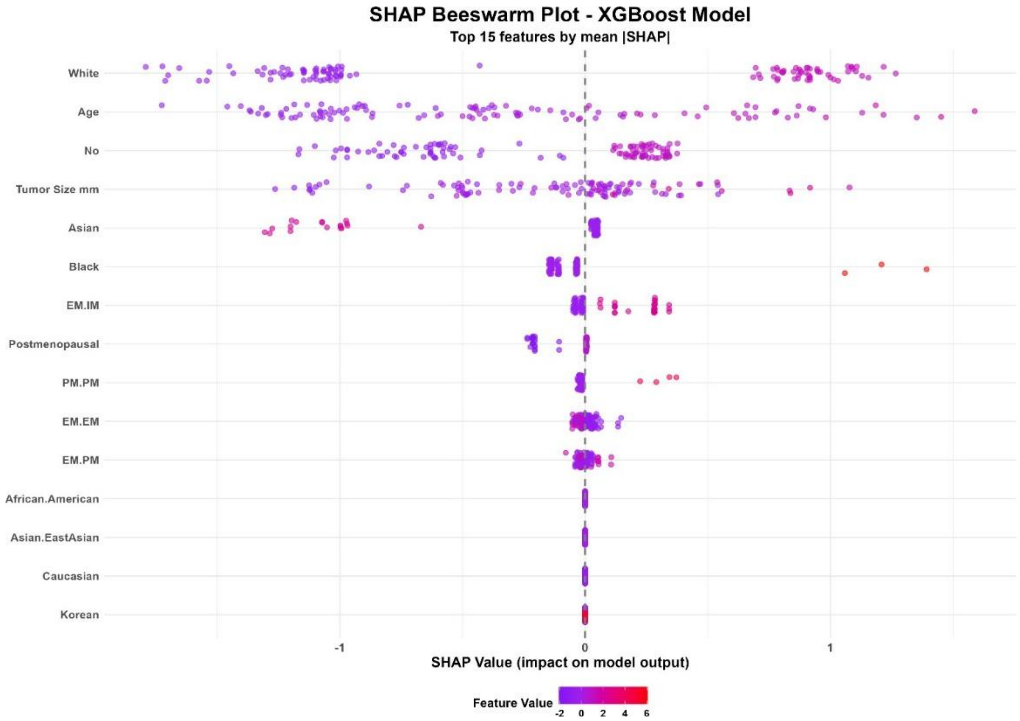

Figure S2. Beeswarm plot of SHAP values

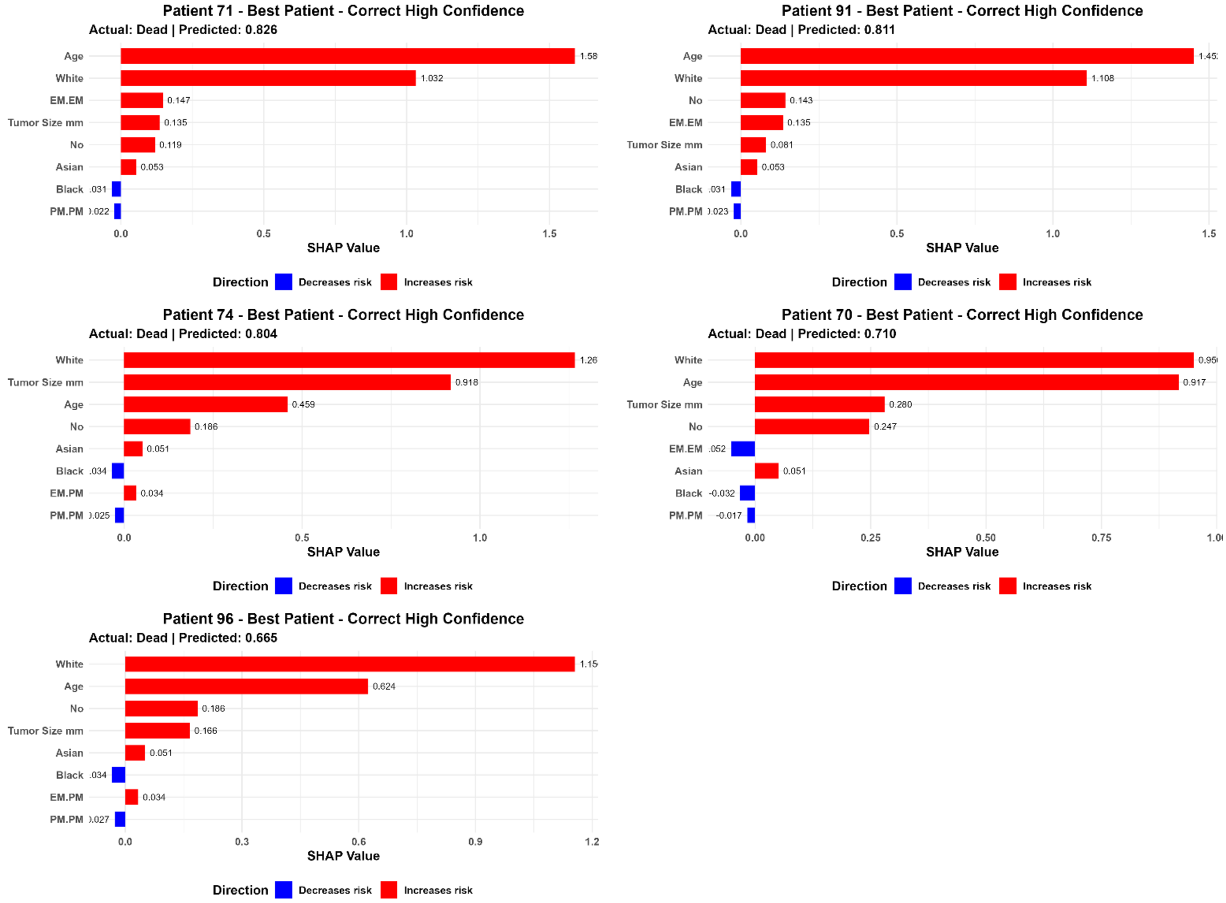

Figure S3. Features according to SHAP values in best 5 patients

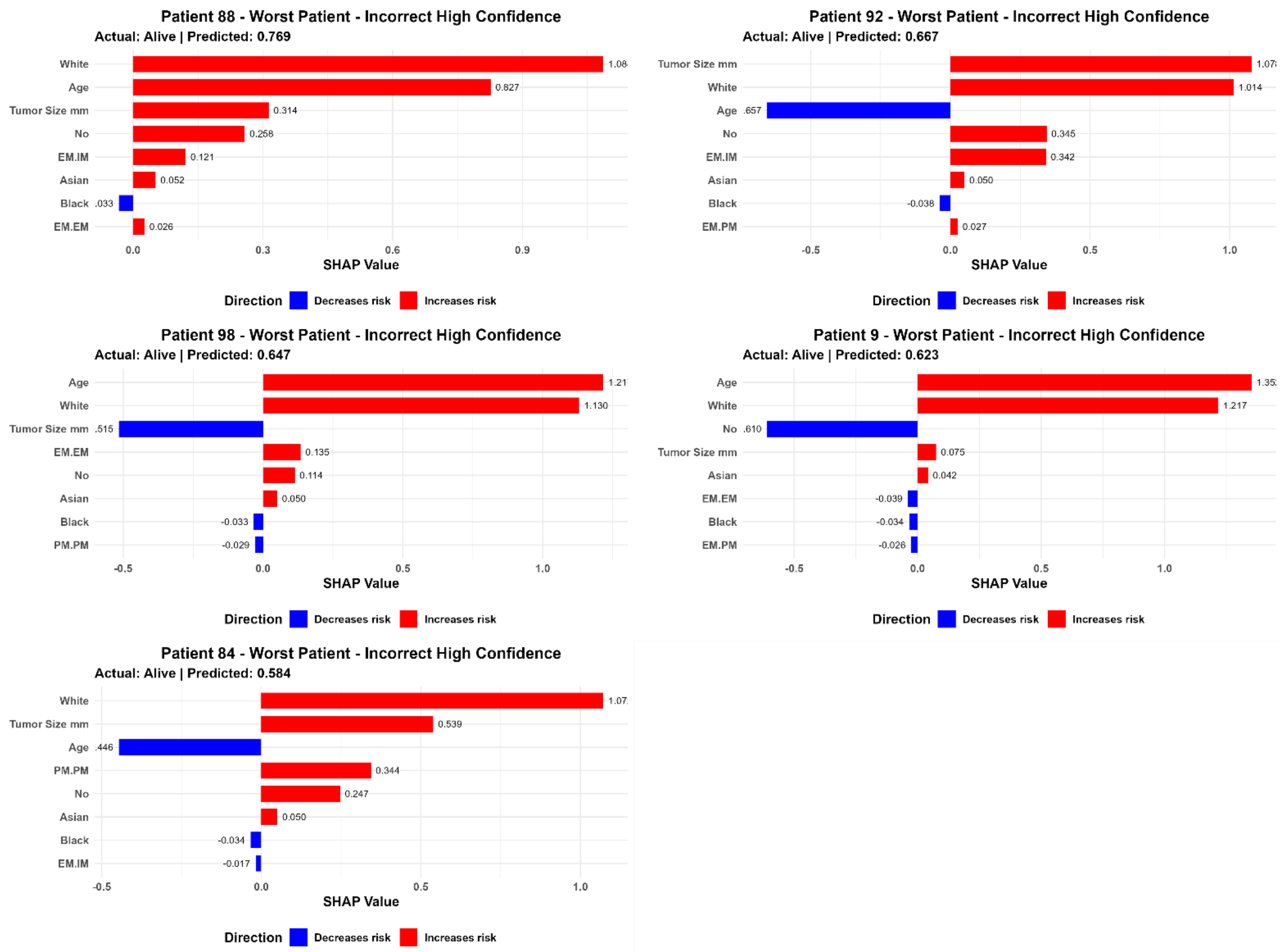

Figure S4. Features according to SHAP values in worst 5 patients

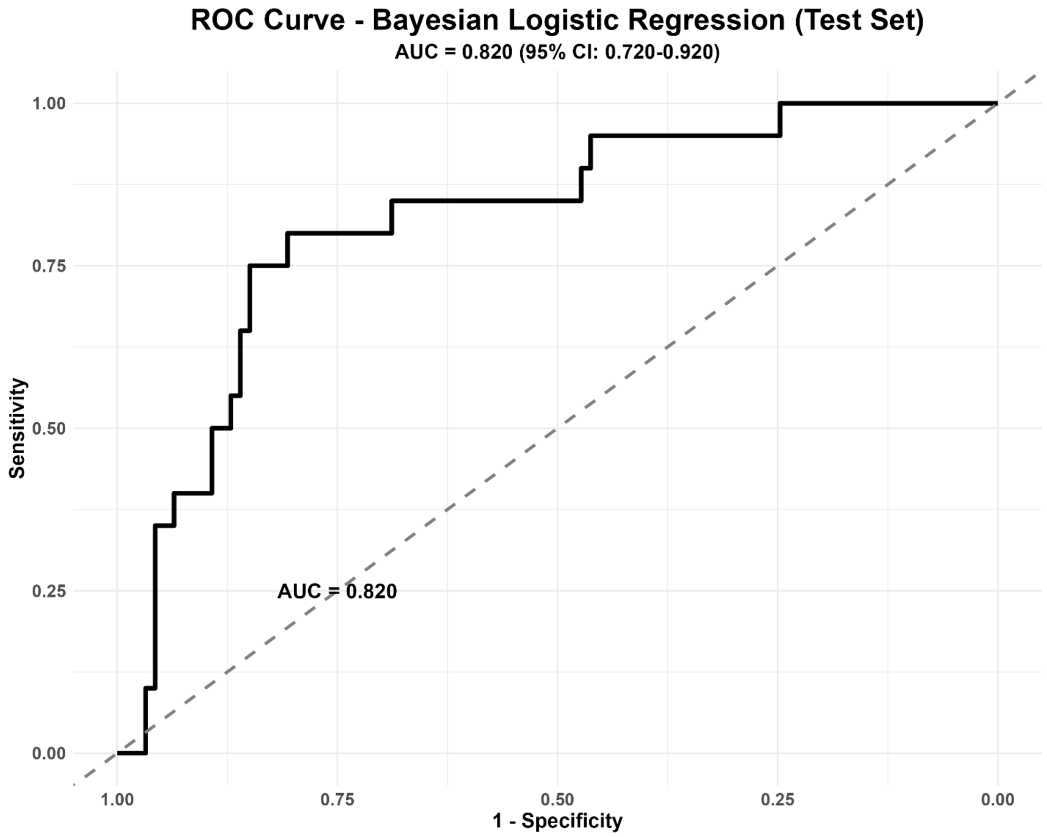

Figure S5. ROC plot of Bayesian logistic regression analysis

CC BY

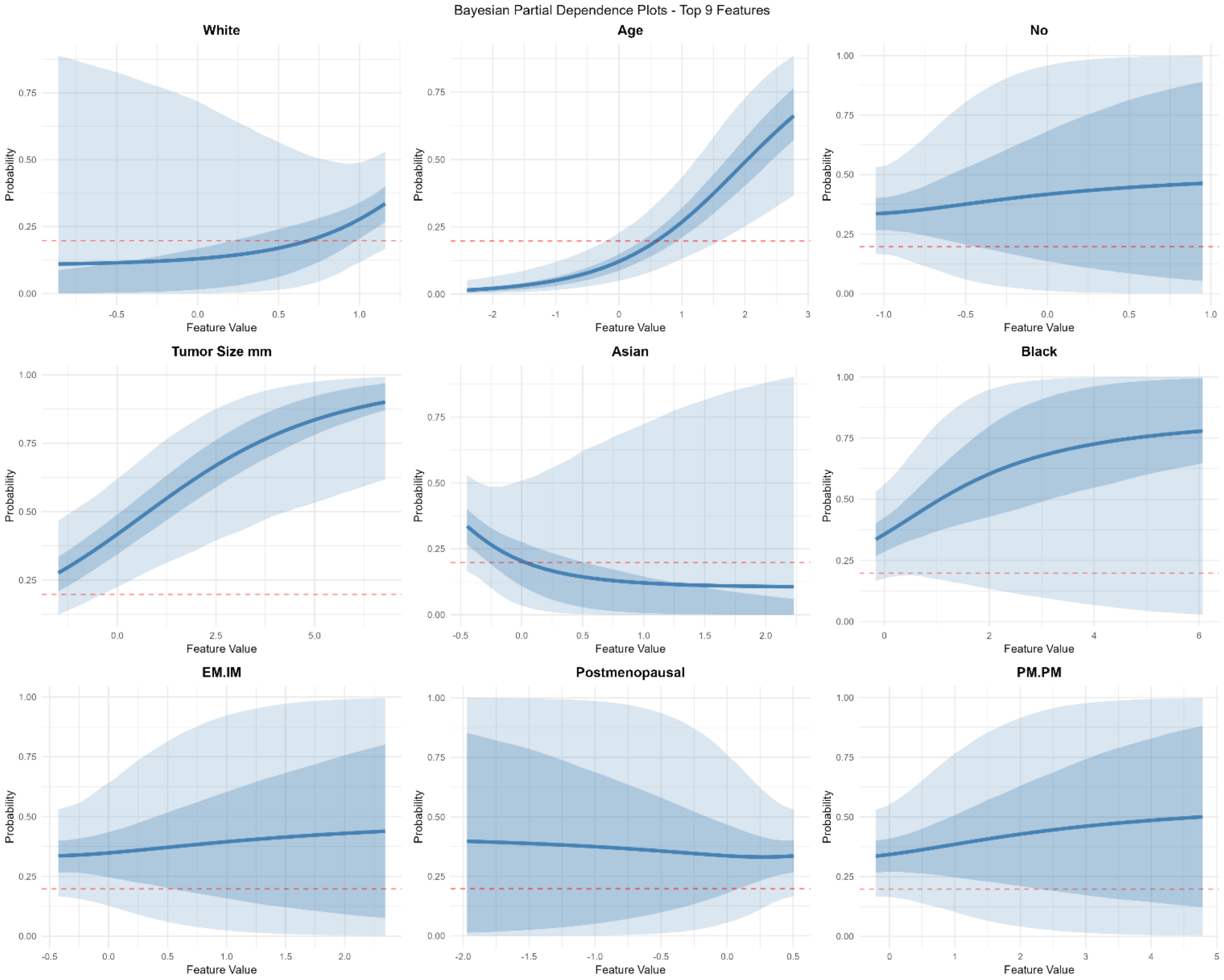

Figure S6. Partial dependence plots of variables in predicting mortality with Bayesian logistic regression analysis
